# A Thermodynamic Analysis of the Binding Specificity between Four Human PDZ Domains and Eight Host, Viral and Designed Ligands

**DOI:** 10.3390/biom11081071

**Published:** 2021-07-21

**Authors:** Eva S. Cobos, Ignacio E. Sánchez, Lucía B. Chemes, Jose C. Martinez, Javier Murciano-Calles

**Affiliations:** 1 Departamento Química Física, Unidad de Excelencia de Química Aplicada a Biomedicina y Medioambiente, Facultad de Ciencias, e Instituto de Biotecnología, Universidad de Granada, 18071 Granada, Spain; evasan@ugr.es (E.S.C.); jcmh@ugr.es (J.C.M.); 2 Laboratorio de Fisiología de Proteínas, Facultad de Ciencias Exactas y Naturales, Consejo Nacional de Investigaciones Científicas y Técnicas, Instituto de Química Biológica de la Facultad de Ciencias Exactas y Naturales (IQUIBICEN), Universidad de Buenos Aires, 1428 Buenos Aires, Argentina; isanchez@qb.fcen.uba.ar; 3 Instituto de Investigaciones Biotecnológicas (IIBiO-CONICET), Universidad Nacional de San Martín, 1650 Buenos Aires, Argentina; lchemes@iib.unsam.edu.ar

**Keywords:** PDZ domain, binding specificity, virus, thermodynamics, isothermal titration calorimetry, differential scanning calorimetry

## Abstract

PDZ domains are binding modules mostly involved in cell signaling and cell–cell junctions. These domains are able to recognize a wide variety of natural targets and, among the PDZ partners, viruses have been discovered to interact with their host via a PDZ domain. With such an array of relevant and diverse interactions, PDZ binding specificity has been thoroughly studied and a traditional classification has grouped PDZ domains in three major specificity classes. In this work, we have selected four human PDZ domains covering the three canonical specificity-class binding mode and a set of their corresponding binders, including host/natural, viral and designed PDZ motifs. Through calorimetric techniques, we have covered the entire cross interactions between the selected PDZ domains and partners. The results indicate a rather basic specificity in each PDZ domain, with two of the domains that bind their cognate and some non-cognate ligands and the two other domains that basically bind their cognate partners. On the other hand, the host partners mostly bind their corresponding PDZ domain and, interestingly, the viral ligands are able to bind most of the studied PDZ domains, even those not previously described. Some viruses may have evolved to use of the ability of the PDZ fold to bind multiple targets, with resulting affinities for the virus–host interactions that are, in some cases, higher than for host–host interactions.

## 1. Introduction

Protein–protein interactions are crucial in biological functionality and are usually organized in networks that comprise two distinctive features: the occurrence of hubs and the repetitiveness of modules across the interactome. Precisely, hub proteins are commonly modular, which confers them the faculty to interconnect multiple processes by interacting with diverse partners. One of the most frequent modules present in hub proteins, and, possibly, the best studied to date, is the PDZ domain. More than 270 PDZ domains are part of human proteins and their principal roles are related to cell–cell communication and signaling and cell polarity [1]. These domains are especially relevant in the synaptic trafficking [2], as well as the tight junction formation and the maintenance of tissue integrity [3]. These functions make the PDZ domain very appealing to viruses, due to the importance for entrance and/or egression [4,5]. Indeed, plenty of viruses act through a PDZ-mediated interaction and PDZ domains are considered as a common route used by pathogenic viruses [6].

Another plausible good reason for virus to interact with the PDZ module is the relatively simple mode of interaction of PDZ binding. PDZ domains typically bind the C-terminal regions of their protein targets, which are often structurally disordered. In this canonical interaction, the residue comprising the free carboxyl terminus is accommodated in a conserved region of the PDZ domain. The residue at the C-terminus is frequently hydrophobic and is numbered as position 0 in the regular PDZ ligand notation (the rest of the residues are numbered backwards: -1, -2, etc.). The binding affinity and, therefore, specificity, in a given PDZ domain, is determined by the amino acid sequence of the ligand. Indeed, traditionally, PDZ domains have been classified by the sequence patterns that are able to recognize:

- Class I: X-(S/T)-X-ϕ_-COOH_;
- Class II: X-ϕ-X-ϕ_-COOH_;
- Class III: X-(D/E)-X-ϕ_-COOH_.

where X is any amino acid, ϕ is a hydrophobic residue and the subscript _-COOH_ means the C-terminus with the free carboxylate. This classification may be called “classic” or “traditional”, since other attempts have been conducted in order to expand and make more precise the specificity classes. For instance, Tonikian et al. described up to 16 specificity classes based on peptide phage display screening, where specificity was determined down to the residue at position -5 [7]. Among them, Luck et al. reviewed the cases where a more extended surface and other structural regions different to the binding pocket modulate PDZ binding [8]. Moreover, Amacher et al. have summarized all the existing PDZ-binding data in a recent review [9]. In any case, the determinants of binding specificity are yet to be uncovered and the more basic and traditional classification has still validity.

Due to the magnitude and significance of viral infections worldwide and taking into account that plenty of diverse viruses bind PDZ domains as a part of their mechanisms of infection, proliferation and/or egression, we decided to perform a comparative study on PDZ binding to viral and host targets, along with designed ligands. Host PDZ motifs and viral mimics differ in many cases in sequence beyond the motif-determining residues. These may lead to differences in the specificity and thermodynamics of virus–host and host–host, protein–protein interactions [10]. Herein we have selected four PDZ domains, three of them known to be hijacked by viruses. Our selected domains comprise the three “classic” binding specificity classes. We have chosen natural ligands from the host and also from viruses that either are described to bind our selected PDZ domains or not, as well as a designed binder for one of the selected domains. Covering the whole set of cross interactions between the domains and the ligands using calorimetric techniques, we have obtained thermodynamic information for the cognate interactions and for non-cognate partners that also present measurable binding. Putting together all our information and gathering additional thermodynamic information from the literature, we shed some light on the intricate jungle of PDZ specificity.

## 2. Materials and Methods

### 2.1. PDZ Domains Purification and Peptide Samples

The DNA encoding the sequences of PSD95-PDZ2 (residues 155–249, according to PSD95 numbering), PSD95-PDZ3 (residues 302–415, according to PSD95 numbering), nNOS-PDZ (residues 1–127, in nNOS numbering) and Erbin-PDZ (given generously by Prof. Sachdev Sidhu, University of Toronto, Toronto, ON, Canada), were sub-cloned into pBAT4 vector (EMBL Core Purification Facility, Heidelberg, Germany) and expressed in Escherichia coli BL21-DE3 cells. Protein purification was carried out as previously described [11,12]. Briefly, cultures were over-expressed overnight and centrifuged. After cell lysis with a French press, the lysates were centrifuged and the pH of the supernatant was lowered down to a pH of 3 with diluted HCl. Upon centrifugation, ammonium sulfate was added to the samples, which were then injected into an AKTA FPLC for preparative size exclusion chromatography. Roughly 15 milligrams of protein per liter of LB culture were obtained and purity was determined by SDS-Page electrophoresis. MALDI-TOF spectrometry, performed at the Scientific Instrumentation Services (CIC) of the University of Granada (Granada, Spain), confirmed the molecular mass for each PDZ domain. Extinction coefficients, determined as described [13], were used to calculate sample concentration. Experimental samples were always prepared by extensive dialysis against 50 mM potassium phosphate buffer at a pH of 7.5 and 4 °C.

Peptide samples were purchased both in JPT peptide technologies (Berlin, Germany) and in ChinaPeptides (Shanghai, China). In the cases where no Tryptophan or Tyrosine residue were present in the peptides, a quantitative analysis was performed and the extinction coefficients were experimentally obtained at 200 nm: 6458 M^−1^·cm^−1^ (for FRETEV), 3272 M^−1^·cm^−1^ (for IAESSV), 4160 M^−1^·cm^−1^ (for KIATLV), 5251 M^−1^·cm^−1^ (for GLDVPV), 9108 M^−1^·cm^−1^ (for HRNTVV), 6184 M^−1^·cm^−1^ (for LLDEIAV) and 9342 M^−1^·cm^−1^ (for VVKVDSV).

### 2.2. Isothermal Titration Calorimetry (ITC)

Calorimetric titrations of the PDZ domains with the peptides were undertaken at 25 °C on an ITC-200 titration microcalorimeter (Microcal Inc., Northampton, MA, USA), as previously described [14]. Protein concentrations in the cell were routinely 70 μM and peptides solutions were prepared using the dialysis buffer to have a final concentration of 900 μM. Titrations were all made by injecting 2 μL volumes of ligand solution, with a final number of 19 injections per experiment. As a control and reference, independent experiments were carried out with only buffer in the calorimeter cell with the same ligand solutions to determine the corresponding heat dilution. The dilution isotherms were then subtracted from that obtained for protein titration. The binding stoichiometry, n, the dissociation constant, K_d_ and the corresponding enthalpy change, ΔH, were determined by fitting the resulting titration isotherm to a model considering a single ligand binding site using ORIGIN 7.0 (OriginLab Corporation, Northampton, MA, USA, 2002).

### 2.3. Differential Scanning Calorimetry (DSC)

Experiments were carried out in a VP-DSC instrument from Microcal INC. as described elsewhere [11]. The buffer conditions were the same as with ITC experiments, 50 mM buffer potassium phosphate at a pH of 7.5. For experiments including the peptides, the final concentration ratio was 1:3 (protein:peptide). Final data were obtained after treatment with ORIGIN 7.0 (OriginLab Corporation, Northampton, MA, USA, 2002).

## 3. Results

### 3.1. Rationale of the Choice of the PDZ Domains and Binding Partners

Among the plethora of the virus-interacting PDZ domains, we decided to select four domains that would allow us to address selectivity and specificity in terms of their respective partners and the canonical specificity classes. Firstly, we chose the highly studied PSD95 s and third PDZ domains. PSD95 is well known to act as a hub protein that interacts with multiple partners, allowing the synaptic trafficking [2]. PSD95 is just hijacked by two viruses: Human Papillomavirus (HPV) and Human T-cell leukemia/lymphotropic virus (HTLV). There is a homolog of PSD95, DLG1 or SAP97, which is bound by multiple viruses (more than 5 kinds, see [6]). We therefore opted for PSD95, to confirm a more built-in selectivity. We have selected its second and third PDZ domains, which are known to have similar specificities, both belonging to class I, although each one exerts diverse functions [15]. Our selection pursues to know whether the interaction energetics influences or reflects selectivity differences among the domains and between the viral ligands. Accordingly, we decided to pick the HTLV virus, since HPV has been demonstrated to be more promiscuous, as it targets several PDZ-containing proteins (at least 13 proteins have been described thus far [6]). There are several HTLV virus species and we opted to choose HTLV-1 and HTLV-3, since both are known to interact with PSD95 [16,17]. Additionally, we selected Ad E4 ORF adenovirus as a plausible negative control, since it should not bind PSD95, nor the other PDZ domains chosen in this study [6].

Secondly, we chose the Erbin PDZ domain, which, interestingly, has been shown to bind both class I [18] and class II [19] ligands. In this case, our choice was based more on the host partners than the viral, although just one virus is known to interact with Erbin, HTLV-1 [20], the one that we selected for PSD95 interaction. Regarding host interactions, we just mentioned that PSD95 regulates neuronal signaling. An important class of PSD95 partners are the tyrosine kinases that trigger several signaling cascades upon activation by neuregulin [21]. These tyrosine kinases, which act as receptors, are, among others, ERBB2 and ERBB4 [22]. Remarkably, ERBB2, whose C-terminus belongs to PDZ specificity class II, does not interact with PSD95, but does bind Erbin [19], whereas ERBB4 (specificity class I) interacts with PSD95 but not with Erbin [19,22]. We then considered to be interesting choosing the C-terminal regions of both receptors. In addition, we also selected a designed peptide for Erbin-PDZ, with the sequence TGWETWV. This peptide was obtained after phage display selections [23] and has served for other specificity studies [7,24,25]. As it can be inferred by the sequence, the designed peptide belongs to specificity class I.

Thirdly, we selected the nNOS PDZ domain, since it may be considered as one of the most versatile PDZ domains in terms of binding [26]. In the early studies of PDZ binding, the characteristic nNOS-PDZ interaction was its internal recognition by both the PDZ domain of syntrophin [24] and PSD95-PDZ2 [25,27]. Nevertheless, more interactions have been shown for nNOS-PDZ, comprising all the three PDZ classes ligands. As cognate nNOS partners we selected a described class II peptide, the C-terminus of CAPON, a cytoplasmic protein that regulates the PSD95/nNOS interaction by competing with PSD95 for nNOS binding [28]. In addition, we chose a class III peptide known to bind nNOS-PDZ, MelR, the C-terminal region of a melatonin receptor [29]. Interestingly, nNOS has not been described to be hijacked by any virus, so its ability to bind a significant diversity of targets, with its important neuronal role, together with PSD95 (with whom it indeed interacts) and Erbin, prompted us to choose this PDZ domain as well.

**Figure 1:**
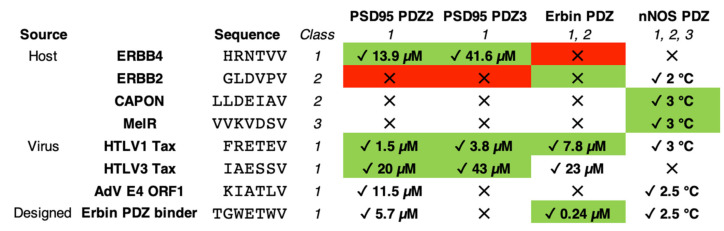
The PDZ domains and the ligands used in this study. The ligands are grouped by their sources (host, virus or design) and are noted with both their respective names and amino acid sequences. The color fill means that the interaction has been previously described (green), or has also been demonstrated not to occur (red). If there is no fill, the interaction has not been studied according to bibliography, to the best of our knowledge (the references for the described interactions are in the text). The tick (✓) and the cross (✕) signs mean whether there is interaction or not, respectively, as demonstrated in this study. We have included the K_d_ in μM or the ΔT_m_ in °C, whenever an interaction has been shown by ITC or DSC, respectively (see Section 3.2 and Section 3.3 for details).

Thus, our selection comprises all three canonical and traditional binding classes of PDZ domains, including viral, host and designed ligands. All our selected PDZ domains and partners are summarized in Figure 1. Remarkably, basically all known interactions between PDZ domains and viruses are based on class I interactions (see Table 1 in [4]). All our selected PDZ domains also share relevant roles in neurons and synaptic trafficking.

### 3.2. ITC Experiments Between the PDZ Domains and Their Ligands

**Figure 2:**
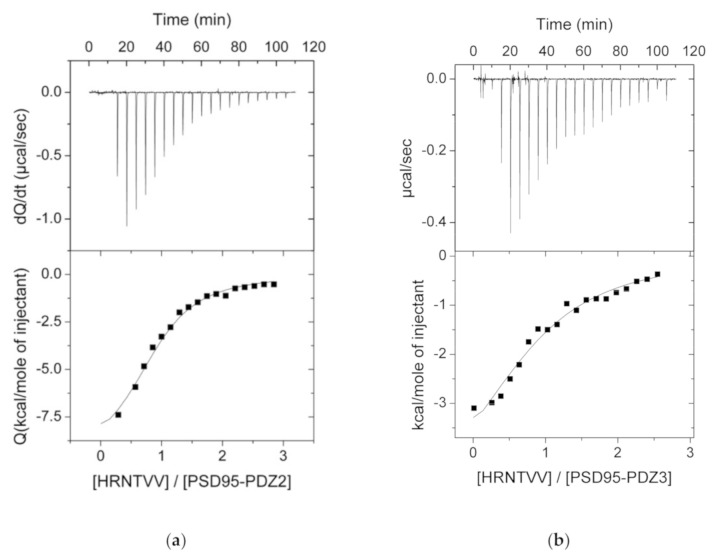

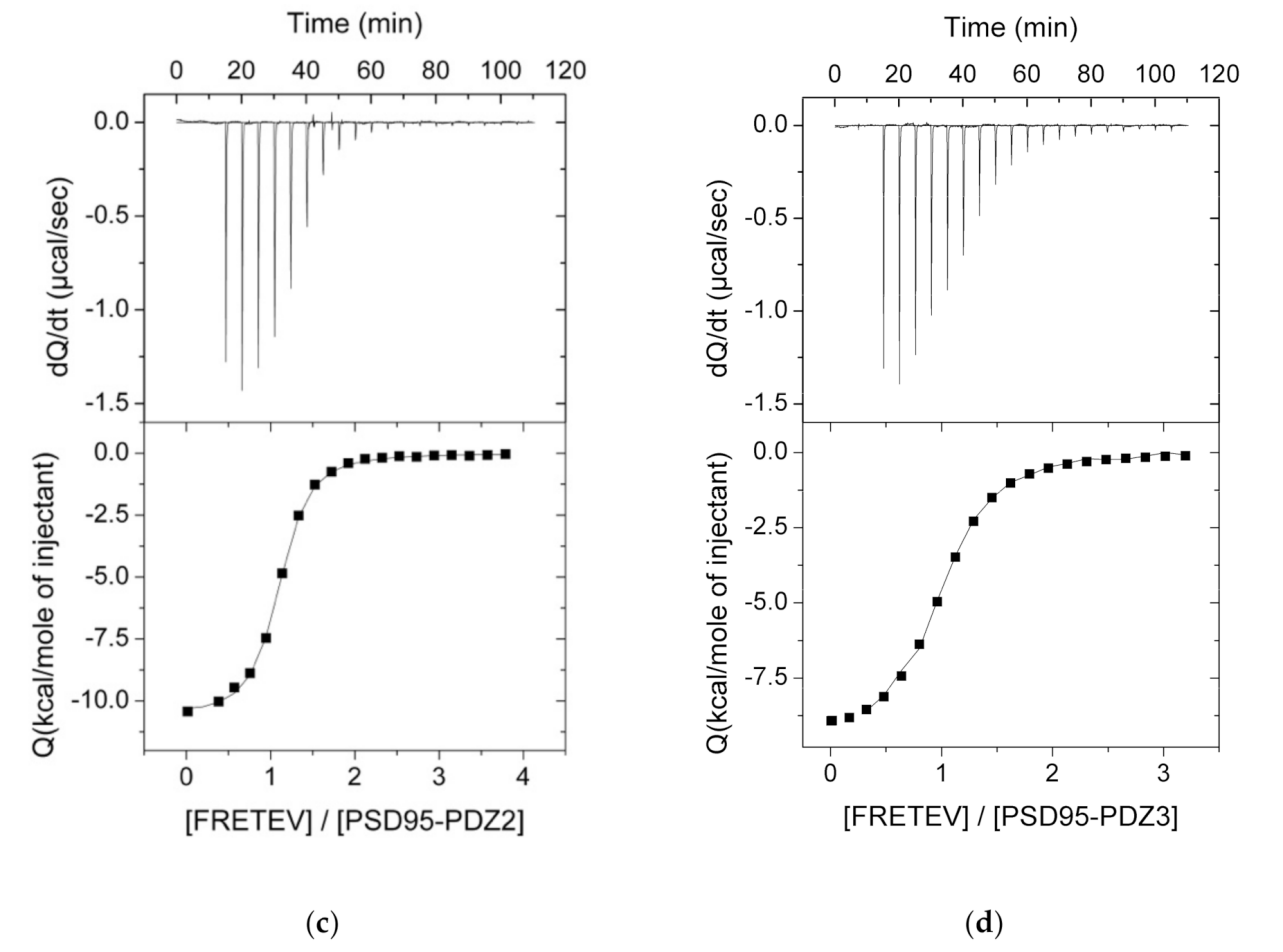
ITC experiments showing previously described interactions between the PDZ domains of PSD95 and both host (ERBB4) and viral (HTLV-1) ligands. Each panel corresponds to: (**a**) HRNTVV (ERBB4) with PSD95-PDZ2; (**b**) HRNTVV (ERBB4) with PSD95-PDZ3; (**c**) FRETEV (HTLV-1) with PSD95-PDZ2; (**d**) FRETEV (HTLV-1) with PSD95-PDZ3.

We performed ITC experiments at 25 °C between all the selected PDZ domains and peptides. For the sake of comparison, the experiments were all conducted in the same buffer conditions, 50 mM potassium phosphate and pH of 7.5, which presents the advantage of having an insignificant protonation heat, meaning that the experimentally determined enthalpy changes should just account for the net heat of reaction [14,30]. A table summarizing all interactions is shown in Figure 1, whilst we have just included the successful experiments graphs, both in Figure 2 and in the SI (Figure S1). As expected, many of the experiments showed no binding, since each PDZ domain has their corresponding partners previously described (see Figure 1).

PSD95-PDZ2 showed binding with all the peptide ligands that belong to class I specificity. K_d_ ranged from 1.5 to 20 μM and ΔH values were between -5.5 and -11.9 kcal·mol^−1^ (Table 1, see Section 3.3 for a dissection of the thermodynamics of interaction). Seemingly, according to these results, PSD95-PDZ2 is quite promiscuous, since only peptides belonging to classes II and III are unable to bind this domain. Maybe the most striking ligand partner of PSD95-PDZ2 from those analyzed is the designed binder for Erbin-PDZ. The interaction with it is moderately strong (5.7 μM), just one order of magnitude below than that for Erbin-PDZ (0.24 μM, Table 1).

Interestingly, results are different for PSD95-PDZ3. The K_d_ values are slightly higher than those obtained for PSD95-PDZ2 (range 3.8–43.8 μM), with smaller enthalpy changes as well, but, more importantly, binding has not been detected for two of the class I peptides (Figure 1), including the designed peptide for Erbin-PDZ. Again, no class II or III peptides were able to bind PSD95-PDZ3 either. A similar selectivity is found for Erbin-PDZ, with the notorious difference of its designed ligand (the tightest binder in this study, K_d_ = 0.24 μM and ΔH = −14.9 kcal·mol^−1^). Instead, the striking result of Erbin-PDZ is the lack of binding with its canonical host partner, ERBB2, which actually gives the name to Erbin: ERBB2-interacting protein [31]. Our result may be due to the length of our peptide. We selected six residues as a standard for the recognition of the C-terminus of a PDZ domain partner, since, according to Tonikian et al., after position −5 (position 0 is the C-terminus and then numbering goes backward), PDZ domains are not specific enough [7]. In the case of ERBB2, it was already reported that the C-terminal tail comprising 9 residues (EYLGLDVPV) only binds with a K_d_ of around 50 μM and the same peptide with the tyrosine phosphorylated has a K_d_ of around 128 μM [32]. Thus, noting the relevance of the tyrosine residue at position −7, we postulate that shortening of the peptide might have caused the absence of detectable binding through ITC.

In the case of nNOS, we did not find binding using ITC with any of the peptides of our study. For all the non-binding ITC experiments, we then performed DSC in the search for weaker affinities binders (see following section).

### 3.3. DSC Experiments for the ITC That Did Not Show Binding

With those binding experiments that did not reveal an interaction by ITC, we decided to test them with DSC, both with and without the ligands, in the same buffer conditions. The final ratio of PDZ:peptide was always 1:3. DSC is also a calorimetric technique but that allows finding a more subtle binding, weaker than that from ITC [33]. Undoubtedly, ITC presents greater advantages, since it gives an exhaustive characterization of the binding interaction, granting the whole thermodynamics of the system; nevertheless, if the interactions are out of the ITC range, usually when they are weak (Kd values in the mM range), it is not sensitive enough to detect binding. By contrast, DSC can detect weak binding by a displacement of the melting temperature (T_m_) due to the stabilization that occurs upon binding. Essentially, when a ligand is bound to the protein, energy is needed to decouple the interacting pair before protein denaturalization. In this way, the T_m_ is increased and the ΔT_m_ is proportional to the interaction energy.

**Figure 3:**
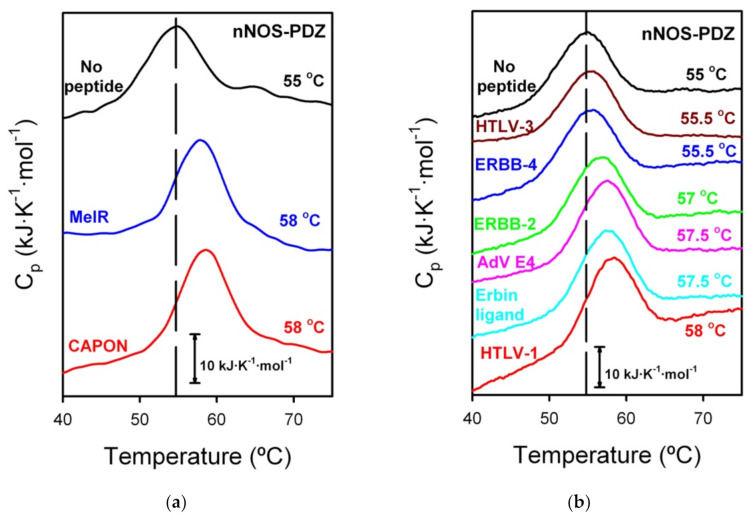
DSC experiments of nNOS-PDZ with the eight peptides of this study. In both panels, the nNOS-PDZ denaturalization without any peptide is also shown as reference, in black. We made two sets of experiments, (**a**) with cognate ligands and (**b**) with non-cognate ligands, including, in both sets, the nNOS-PDZ alone. The T_m_ is noted next to each calorimetric trace in the same color code (“Erbin ligand” refers to the phage-display designed ligand for Erbin). A bar indicating 10 kJ·K^−1^·mol^−1^ is depicted as a reference.

We therefore tested our failed-ITC PDZ-ligand experiments using DSC. Remarkably, no binding was shown for any of the PDZ domains, except for nNOS-PDZ (Figure 3). Thus, PSD95-PDZ2, PSD95-PDZ3 and Erbin-PDZ seem to present stronger binding with their partners and no binding with others, reflecting a more stringent selectivity.

DSC traces showed a T_m_ displacement in the case of nNOS-PDZ, with five of the tested ligands of around 2–3 °C. Unfortunately, the calorimetric traces for nNOS-PDZ are highly irreversible [12], which prevents an analysis that would potentially yield a K_d_ value. Instead, we can approximately estimate a K_d_ range based on previous cases in PDZ binding. We have taken as a reference two PDZ-peptide interactions. Firstly, the binding of PSD95-PDZ3 with the peptide KKETAV formerly described by us [11,34]. We had previously performed both ITC and DSC with PSD95-PDZ3 and KKETAV. The concentrations in the ITC experiments were the same than those used in this study and the DSC concentrations were similar, with the PDZ:peptide ratio also being 1:3. For the PSD95-PDZ3/KKETAV interaction, a ΔT_m_ of about 9 °C corresponds to a K_d_ of 1.5 μM. In the current study, we have also performed DSC for the interaction between PSD95-PDZ2 and TGWETWV and a ΔT_m_ of around 6 °C corresponds to a K_d_ of 5.7 μM (Table 1 and Figure S2). Considering that they are different PDZ domains, but have a somewhat similar ΔT_m_-K_d_ correspondence, a ΔT_m_ of 2–3 °C would then imply a K_d_ of high micromolar or low millimolar, the range where ITC is not sensitive enough to detect binding. Lower ΔT_m_ values would denote even weaker affinities, out of the commonly accepted values for a physiological or biological interaction. nNOS-PDZ would therefore have affinity in the order of biological interaction with its natural counterparts (CAPON and MelR, specificity class II and III, respectively, ΔT_m_ = 3 °C for both peptides) and, surprisingly, with two C-terminal tails of virus proteins (HTLV1 Tax, ΔT_m_ = 3 °C and AdV E4 ORF1, ΔT_m_ = 2.5 °C, both belonging to class I), host partners (ERBB2, ΔT_m_ = 2 °C, class II) and even the designed peptide for the Erbin-PDZ (ΔT_m_ = 2.5 °C, class I) (see Figure 1). We considered no binding with HTLV-3 and ERBB-4 peptides, since the ΔT_m_ is too low (0.5 °C) and it may be within the experimental error, since these traces are highly sensitive to protein concentration, which allowed to reveal an intermediate in the conformational equilibrium [12]. These results imply that, although not with high affinities, nNOS-PDZ seems to be considerably promiscuous, comprising binding with ligands belonging to all the three traditional specificities in PDZ domains.

### 3.4. Analysis of the Energetic Balance of the Interactions

**Figure 4:**
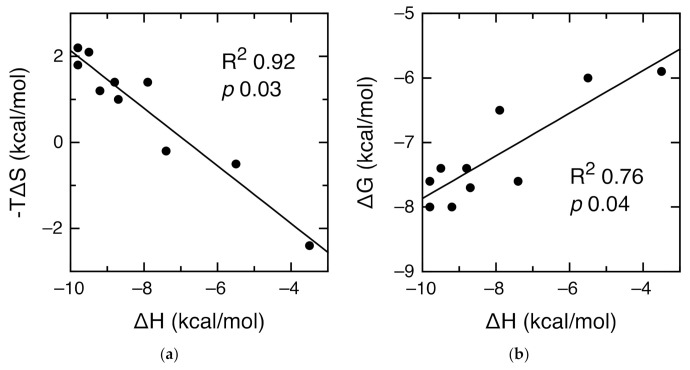
Enthalpy–entropy compensation in the PSD95-PDZ3 interactions with data from this study and from [35]. (**a**) Correlation between -TΔS and ΔH, showing the R^2^ and *p* values. The experimental data are shown in circles and the linear regression as a line, with the equation y = −4.6 − 0.67x. (**b**) Correlation between ΔG and ΔH, showing the R^2^ and *p* values. The experimental data are shown in circles and the linear regression as a line, with the equation y = −4.6 + 0.33x.

We have analyzed the relationship between enthalpy, entropy and free energy of interaction in our ITC results. Since we did not have enough data points for PSD95-PDZ2 or Erbin, we just performed our analysis with PSD95-PDZ3 and included previous data for mutant PSD95-PDZ3 domains measured under the same conditions [35]. Figure 4A shows a linear relationship between both -TΔS and ΔH (R^2^ = 0.92, *p* = 0.03) and ΔG and ΔH (R^2^ = 0.76, *p* = 0.04). Moreover, the slope of the relationship between −TΔS and ΔH is far from unity (−0.67 ± 0.07). Altogether, these observations point at a bona fide enthalpy–entropy compensation phenomenon in the interactions of PSD95-PDZ3 with its targets [36,37,38]. The absence of outliers in the two linear relationships supports a robust mode of interaction that is not fundamentally altered by changes in the sequence of PSD95-PDZ3 or the peptides [39,40]. The slope of the relationship between ΔG and ΔH is 0.33 ± 0.07, indicating that for each energy unit of an increase in interaction enthalpy, one third translates into interaction free energy and two thirds are compensated by entropy [41]. We also analyzed ITC data by Spaller and coworkers for the interaction of PSD95-PDZ3 with six-residue peptides [42,43] and found similar results (Figure S3). In all, the interaction of peptides with PSD95-PDZ3 is robust and shows bona fide enthalpy–entropy compensation.

### 3.5. Comparison With Other PDZ–Ligand Interactions in Bibliography

**Figure 5:**
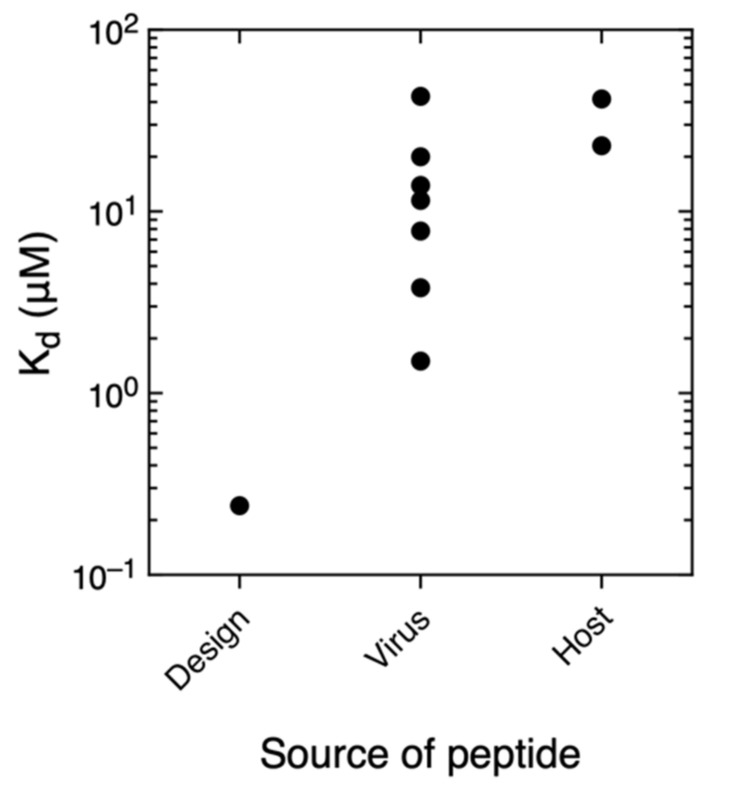
Logarithmic representation of the K_d_ vs. the peptide ligand source in our results.

It has been suggested that viral proteins displace other partners of their host targets by evolving for high affinity [10], so we compared the values of K_d_ depending on the nature and source of the ligand. In Figure 5, we have represented the K_d_ value in a logarithmic scale with respect to the ligand nature (host, virus or design). In our set of PDZ–ligand interactions, the strongest binding is with the designed peptide, then followed by viral ligand interactions and the host interactions as the weakest. This is, in principle, the more intuitive result and we wanted to check if it does happen in other PDZ examples. All the previously described cases, to the best of our knowledge, are represented in graphs, organized by PDZ domain, in the different panels of Figure S4. The trend observed by us happens in PDZ2 and PDZ3 from Scribble interacting with HTLV-1 Tax and host proteins [44], as well as PTPN4-PDZ and MAST2-PDZ with rabies and host proteins [45,46,47,48]. The opposite case appears in MUPP1-PDZ with HPV [49] and PALS1-PDZ with SARS-CoV-2 [50]; the binding affinity between the viral protein and the PDZ domain is weaker than their respective PDZ–host interactions. Finally, for MAGI1-PDZ interaction with HPV E6, HTLV Tax, AdV E4 ORF1 and two host proteins [51], there is no well-defined trend in affinities differences. As a consequence, no clear rule in binding affinity governs the plausible competition between a viral and a host protein, other causes are surely influencing viral infection and proliferation. We speculate the following two scenarios to rationalize this result. Some viral proteins targeting host PDZ proteins show high affinity and function by displacing other host protein targets, thus “hijacking” the host PDZ protein for some specific purpose. Other viral proteins show affinities comparable to other host targets and function by integrating themselves in the existing protein interaction network, thus co-opting the incumbent partners of the host PDZ protein. Additionally, the relative in vivo effective concentrations of virus and host PDZ ligands can also strongly influence binding. Relative effective concentrations may vary during the virus life cycle due to changes in protein expression, localization and conformation. Thus, additional factors should be considered in the future for a definitive description of the competition between virus and host ligands PDZ ligands.

## 4. Discussion

Our results indicate a basic and intrinsic specificity for a single PDZ domain, since all our four examples present binding with some ligands and not with others. However, it is also clear that the degree of such specificity differs in each individual domain. PSD95-PDZ2, PSD95-PDZ3 and Erbin-PDZ bind only class I ligands. Thus, elemental selectivity is present in all three of them. On the other hand, the specificity of the three domains for different class I ligands is different. PSD95-PDZ2 binds all five class I ligands, while PSD95-PDZ3 and Erbin-PDZ bind different sets of three peptide class I ligands. The nNOS-PDZ case is quite interesting, since it does not bind strongly to any of the tested ligands, but it weakly interacts with ligands belonging to the three traditional specificity classes. An alternative would be that the described binding by DSC might be weak enough to be considered as unspecific. Nevertheless, Merino-Gracia et al. dissected the binding specificity in nNOS-PDZ, revealing the molecular determinants of its ability to bind the three described specificity classes [26]. Hence, these authors demonstrated the versatility of this domain to bind multiple and diverse partners by showing at least three slightly different binding modes, including one able to recognize internal motifs and not the free carboxylate of the C-terminus. In several of the binding modes, residues outside the nNOS-PDZ binding pocket are involved and, considering that nNOS is a bigger protein, other additional allosteric pathways may also influence its PDZ binding. Thus, we can assume that our found interactions by DSC are specific and, perhaps, nNOS-PDZ interactions with its partners in vivo might be regulated not by the feeble intrinsic specificity of the domain, but, rather, by other elements, such as allosteric activators or the presence or absence of natural partners due to other signaling cascades.

PSD95-PDZ3 shows, however, a stronger specificity; it is for instance the only PDZ domain in this study that does not bind the phage-display designed binder for Erbin-PDZ. PSD95-PDZ3 has been exhaustively studied and two main factors influence binding: allostery [52,53] and post-translational modifications [35]. In both cases, the α3 helix of PSD95-PDZ3 [54] has a prominent role in the binding regulation, which is extended to the stability and folding of the domain [34,55,56,57]. Interestingly, PSD95 has three PDZ domains, in which PDZ1 and PDZ2 often act as a tandem with a very short linker between them [58,59,60], and PDZ3 alone has its specific functions, where the α3 helix is actually the linker with the next modules of the protein, an SH3 and guanylate-kinase domains. Maybe, the intrinsic binding regulation for PSD95-PDZ3 has then been evolved to be tighter than for PSD95-PDZ2, since the latter relies on its counterpart PSD95-PDZ1 to regulate their functionality. The presence of enthalpy–entropy compensation in the interaction between PSD95-PDZ3 and its targets may be a consequence of this evolutionary constraint.

Specificity regarding the host ligands is well defined. The ERBB4, CAPON and MelR peptides bind only to their respective PDZ domains. As discussed above, the host ERBB2 peptide binds to nNOS-PDZ, but not to its known partner Erbin-PDZ, most likely due to the length of the peptide. Thus, it seems that, at least in our set of PDZ interactions, the host and/or natural PDZ binders are best suited to their own particular domain. The designed and viral ligands have also shown a much larger promiscuity than the host ligands, with just a few cross interactions not taking place. The peptide designed to bind Erbin-PDZ also binds to PSD95-PDZ2 and nNOS-PDZ, which suggests that optimizing affinity can lead to decreased specificity, as discussed before for other PDZ-mediated interactions [61,62].

The viral ligands are also more promiscuous than the host ligands. The HTLV1 Tax peptide binds all four PDZ domains, while the HTLV3 Tax and AdV E4 ORF1 peptides bind three and two out of four PDZ domains, respectively. Our positive results include AdV E4 ORF1, which had not been reported to interact with any of the PDZ domains studied here. In addition, two of the three viral ligands interact with nNOS-PDZ, which has not been described to be the target of any virus up to date. Our results agree with previous high-throughput studies that report the interaction of individual viral proteins with up to dozens of host PDZ domains [63,64,65,66,67,68]. It may be the case that viruses have evolved to sequester a diverse range of host PDZ partners, exploiting the intrinsic interaction plasticity of the PDZ fold [69]. The interaction with multiple functionally related host proteins may increase robustness of the viral life cycle. This is the case for the budding-mediating Ebola virus VP40 protein, which entraps the host ESCRT pathway through several modular domains [70]. We also find it interesting that the homologous Tax proteins from HTLV1 and HTLV3 show differences in binding. This is in agreement with the differences in binding found for homologous papillomavirus E6 proteins and may be related to their clinical phenotypes [67].

Another important consequence of the specificity ligand results in this study is the property of the phage-display designed peptide to bind three of the four PDZ domains of our study and two of them tightly. Certainly, side effects are difficult to avoid, when targeting host proteins that are hijacked by virus. More problems along the same lines can be encountered if a PDZ domain is targeted, since the designed peptide/drug may bind other PDZ domains. Undeniably, designing strong and, more particularly, specific inhibitors for a given PDZ domain poses a significant challenge.

## Figures and Tables

**Table 1:** Thermodynamic parameters of the interactions between the PDZ domains and ligands that have been determined by ITC ^1^.

Source	PDZ	K_d_ (μM)	ΔH (kcal·mol^−1^)	−Τ·ΔS (kcal·mol^−1^)	ΔG (kcal·mol^−1^)
**Host**					
ERBB4	PSD95-PDZ2	13.9 ± 1.4	−9.7 ± 0.3	3.1	−6.6 ± 0.6
	PSD95-PDZ3	41.6 ± 10.2	−5.5 ± 0.1	−0.5	−6.0 ± 1.2
**Virus**					
HTLV1 Tax	PSD95-PDZ2	1.5 ± 0.1	−10.6 ± 0.1	2.6	−8.0 ± 0.5
	PSD95-PDZ3	3.8 ± 0.2	−9.5 ± 0.1	2.1	−7.4 ± 0.5
	Erbin-PDZ	7.8 ± 0.9	−9.3 ± 0.3	2.3	−7.0 ± 0.8
HTLV3 Tax	PSD95-PDZ2	20.0 ± 3.8	−5.5 ± 0.5	−0.9	−6.4 ± 1.3
	PSD95-PDZ3	43 ± 10	−3.5 ± 0.7	−2.4	−5.9 ± 1.9
	Erbin-PDZ	23 ± 9	−2.4 ± 0.3	−3.8	−6.2 ± 1.9
AdV E4 ORF1	PSD95-PDZ2	11.5 ± 0.5	−9.4 ± 0.2	2.6	−6.8 ± 0.3
**Designed**					
Erbin PDZ binder	PSD95-PDZ2	5.7 ± 0.2	-9.6 ± 0.4	2.5	−7.1 ± 0.3
	Erbin-PDZ	0.24 ± 0.08	-14.9 ± 0.1	5.8	−9.1 ± 2.0

^1^ Stoichiometric (*n*) values ranged from 0.8 to 1.1.

## Data Availability

The data presented in this study are available in both this article and its supplementary information.

## Supplementary Materials

The following are available online at https://www.mdpi.com/article/10.3390/biom11081071/s1, Figure S1: ITC experiments of seven PDZ-ligand interactions, Figure S2: DSC experiments of PSD95-PDZ2 alone and with TGWETWV, Figure S3: Enthalpy–entropy compensation in PSD95-PDZ3 interactions with ligands from the literature, Figure S4: Logarithmic representation of K_d_ values of PDZ interactions with virus, host or designed ligands found in bibliography.
