# Supplementary material for: A Thermodynamic Analysis of the Binding Specificity between Four Human PDZ Domains and Eight Host, Viral and Designed Ligands
Source: Biomolecules. 2021 Jul 21;11(8):1071. doi: 10.3390/biom11081071 (PMC8393326; doi:10.3390/biom11081071)
Supplement: Supplementary file 1 [file biomolecules-11-01071-s001.zip › biomolecules-1281405-supplementary.pdf]

Supplementary Material

Specificity and thermodynamics of interaction between four human PDZ domains and eight host, viral and designed ligands

Eva S. Cobos, Ignacio E. Sánchez, Lucía B. Chemes, Jose C. Martínez and Javier Murciano-Calles

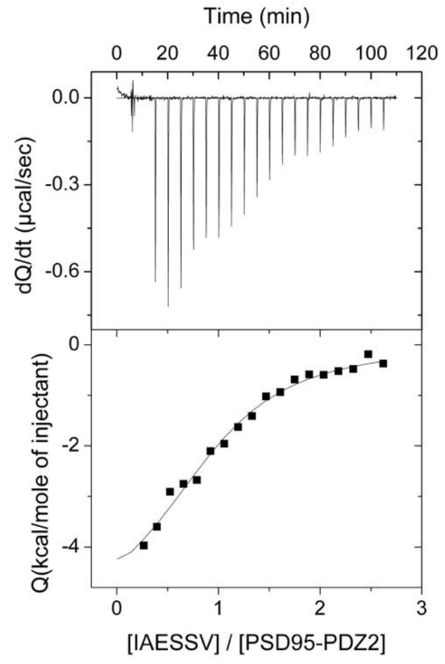

(**a**)

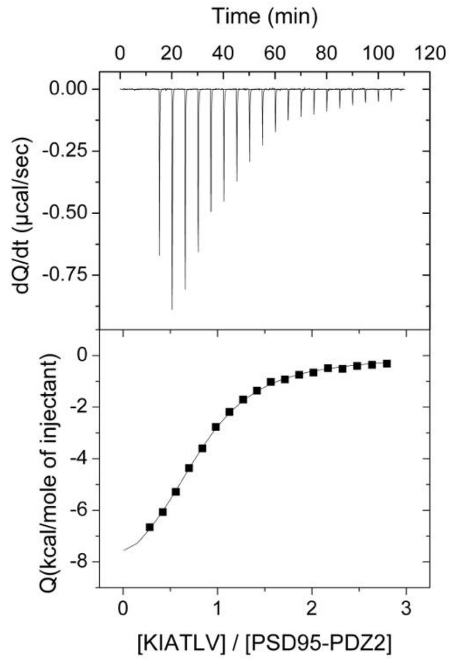

(**b**)

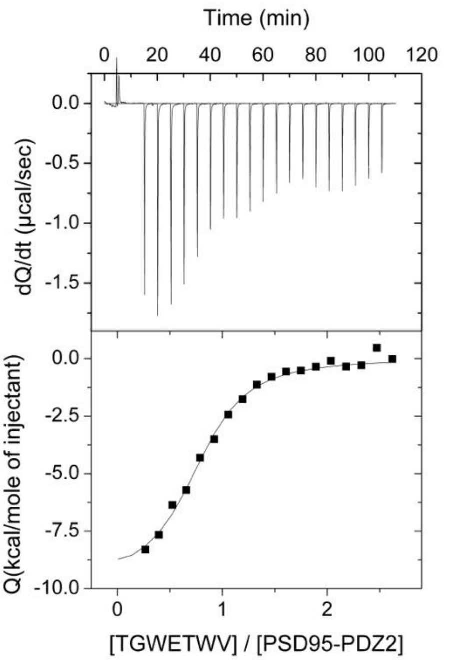
Time (min)
dQ/dt (μcal/sec)
Q(kcal/mole of injectant)
[TGWETWV] / [PSD95-PDZ2]

(**c**)

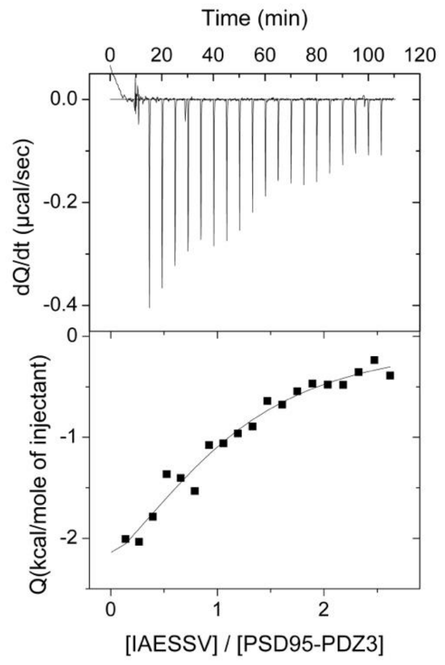
Time (min)
dQ/dt (μcal/sec)
Q(kcal/mole of injectant)
[IAESSV] / [PSD95-PDZ3]

(**d**)

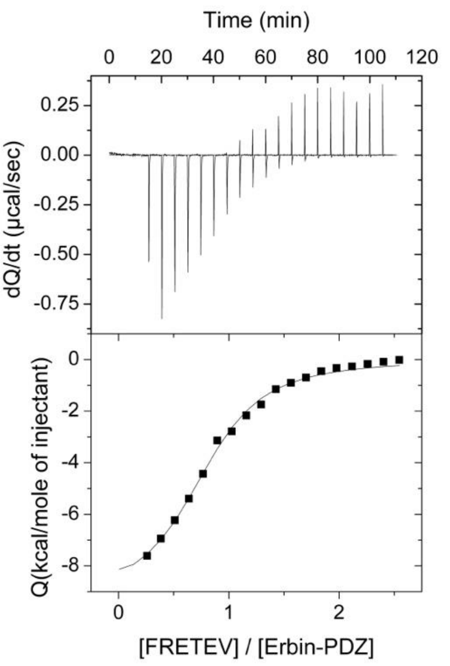
Time (min)
dQ/dt (μcal/sec)
Q(kcal/mole of injectant)
[FRETEV] / [Erbin-PDZ]

(**e**)

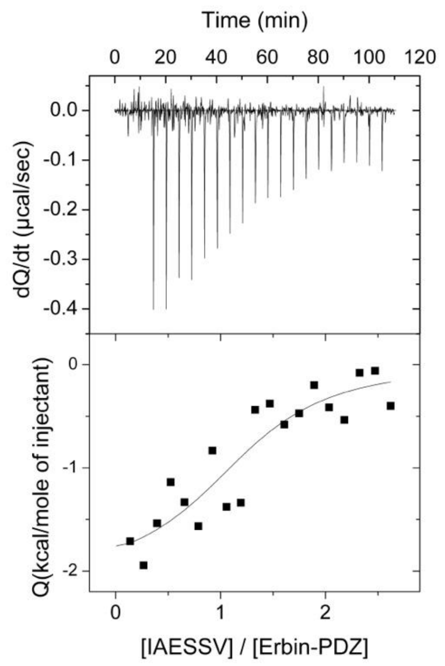
Time (min)
dQ/dt (μcal/sec)
Q(kcal/mole of injectant)
[IAESSV] / [Erbin-PDZ]

(**f**)

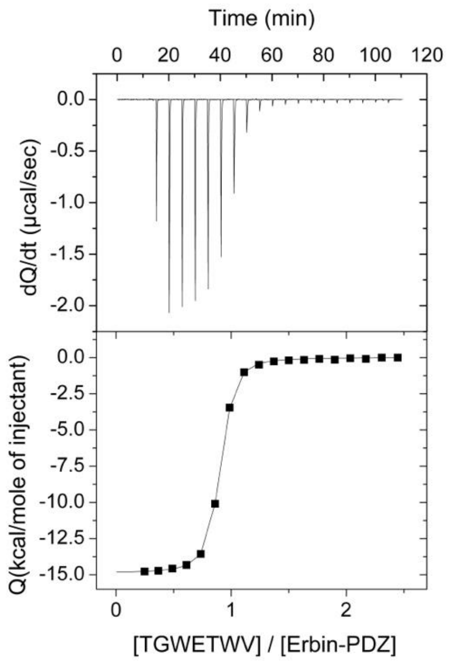

(**g**)

Figure S1. ITC experiments of seven PDZ-ligand interactions: (**a**) PSD95-PDZ2 with IAESSV (HTLV-3); (**b**) PSD95-PDZ2 with KIATLV (AdV E4 ORF1); (**c**) PSD95-PDZ2 with TGWETWV (designed peptide for Erbin-PDZ); (**d**) PSD95-PDZ3 with KIATLV (AdV E4 ORF1); (**e**) Erbin-PDZ with FRETEV (HTLV-1); (**f**) Erbin-PDZ with IAESSV (HTLV-3); (**g**) Erbin-PDZ with TGWETWV (designed peptide for Erbin-PDZ).

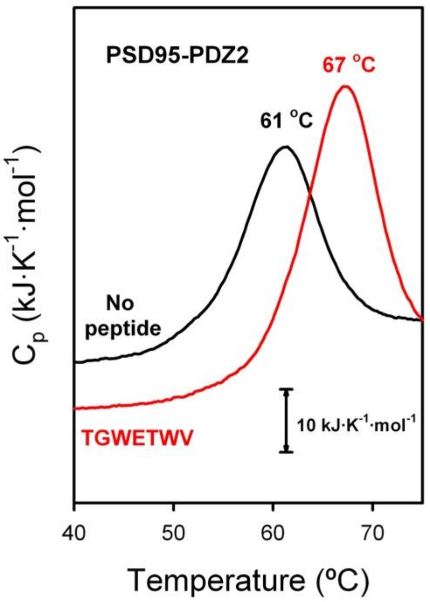

Figure S2. DSC experiments of PSD95-PDZ2 alone and with TGWETWV. The T_m is noted on top of each calorimetric trace in the same color code. A bar indicating 10 kJ·K^{-1}·mol^{-1} is depicted as a reference.

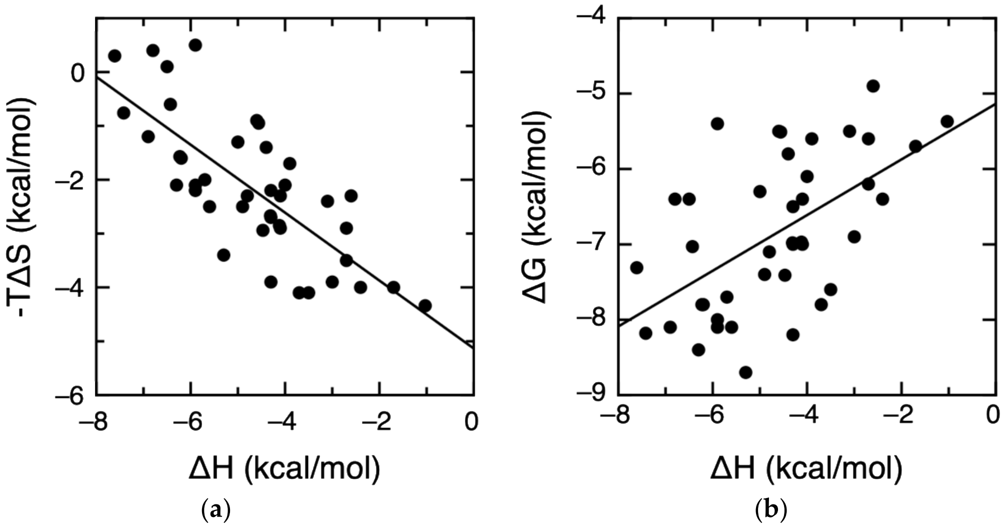

Figure S3. Enthalpy–entropy compensation in PSD95-PDZ3 interactions with ligands from the literature [1,2]. (**a**) Correlation between -TΔS and ΔH. The experimental data is shown in circles and the linear regression as a line, with the equation y = -5.1 - 0.63·x, R^2 = 0.6 and p = 0.003. (**b**) Correlation between ΔG and ΔH. The experimental data is shown in circles and the linear regression as a line, with the equation y = -5.1 + 0.37·x, R^2 = 0.3 and p = 0.11.

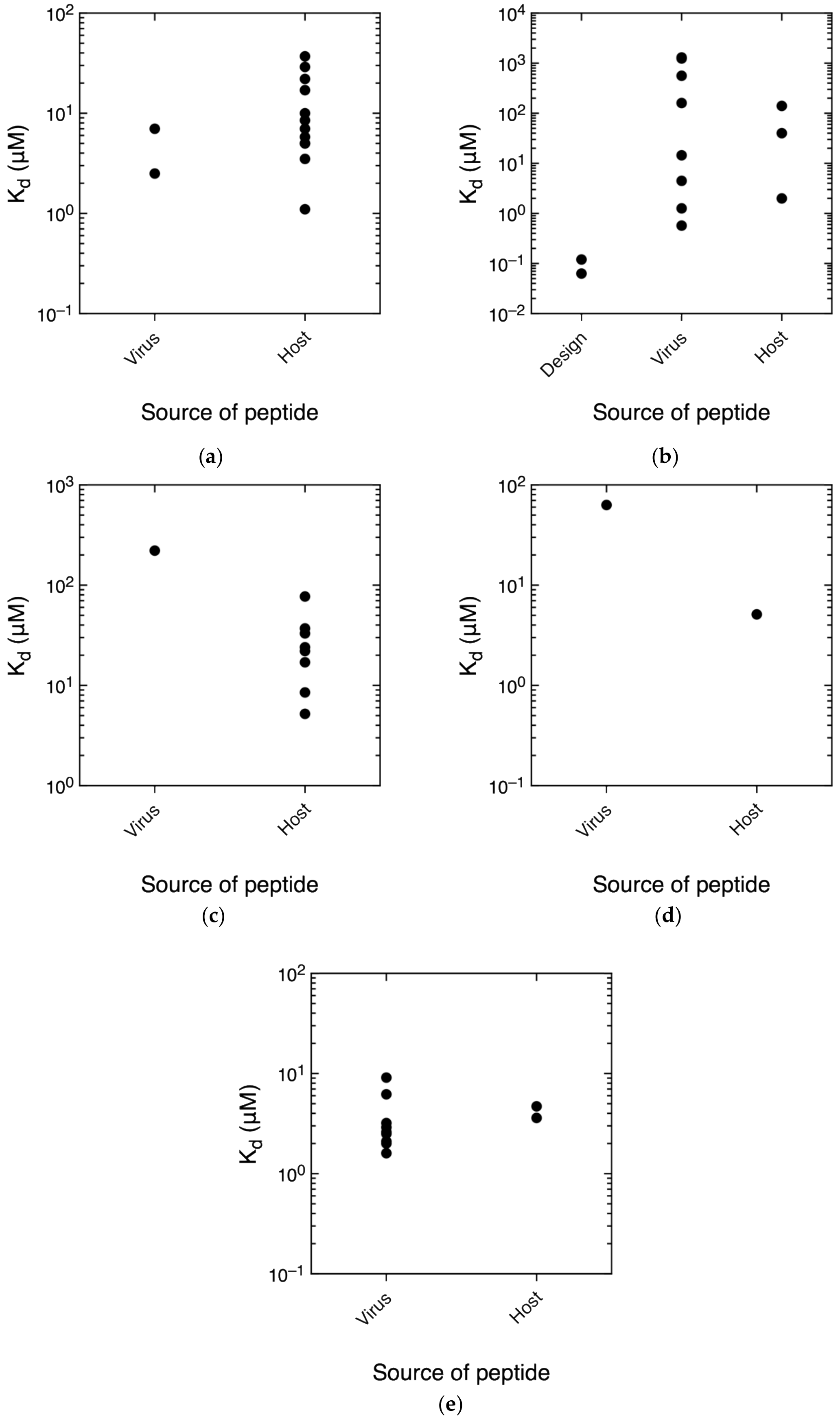

Figure S4. Logarithmic representation of Kd values of PDZ interactions with virus, host or designed ligands found in bibliography. The panels correspond to: (**a**) PDZ2 and PDZ3 from Scribble with HTLV-

1 Tax and host ligands [3]; (**b**) PTPN4-PDZ and MAST2-PDZ with rabies, host and designed ligands [4-7]; (**c**) MUPP1-PDZ with HPV and host ligands [8]; (**d**) PALS1-PDZ with SARS-CoV-2 and host ligands [9]; and (**e**) MAGI1-PDZ interaction with HPV E6, HTLV Tax, AdV E4 ORF1 and two host ligands [10].

1. Saro, D.; Klosi, E.; Paredes, A.; Spaller, M.R. Thermodynamic analysis of a hydrophobic binding site: probing the PDZ domain with nonproteinogenic peptide ligands. *Org Lett* **2004**, *6*, 3429-3432.
2. Saro, D.; Li, T.; Rupasinghe, C.; Paredes, A.; Caspers, N.; Spaller, M.R. A Thermodynamic Ligand Binding Study of the Third PDZ Domain (PDZ3) from the Mammalian Neuronal Protein PSD-95. *Biochemistry* **2007**, *46*, 6340-6352.
3. Ivarsson, Y.; Arnold, R.; McLaughlin, M.; Nim, S.; Joshi, R.; Ray, D.; Liu, B.; Teyra, J.; Pawson, T.; Moffat, J., et al. Large-scale interaction profiling of PDZ domains through proteomic peptide-phage display using human and viral phage peptidomes. *Proceedings of the National Academy of Sciences of the United States of America* **2014**, *111*, 2542-2547, doi:10.1073/pnas.1312296111.
4. Babault, N.; Cordier, F.; Lafage, M.; Cockburn, J.; Haouz, A.; Prehaud, C.; Rey, Félix A.; Delepierre, M.; Buc, H.; Lafon, M., et al. Peptides Targeting the PDZ Domain of PTPN4 Are Efficient Inducers of Glioblastoma Cell Death. *Structure* **2011**, *19*, 1518-1524, doi:https://doi.org/10.1016/j.str.2011.07.007.
5. Delhommel, F.; Chaffotte, A.; Terrien, E.; Raynal, B.; Buc, H.; Delepierre, M.; Cordier, F.; Wolff, N. Deciphering the unconventional peptide binding to the PDZ domain of MAST2. *Biochemical Journal* **2015**, *469*, 159-168, doi:10.1042/bj20141198.
6. Khan, Z.; Terrien, E.; Delhommel, F.; Lefebvre-Omar, C.; Bohl, D.; Vitry, S.; Bernard, C.; Ramirez, J.; Chaffotte, A.; Ricquier, K., et al. Structure-based optimization of a PDZ-binding motif within a viral peptide stimulates neurite outgrowth. *Journal of Biological Chemistry* **2019**, *294*, 13755-13768, doi:https://doi.org/10.1074/jbc.RA119.008238.
7. Terrien, E.; Chaffotte, A.; Lafage, M.; Khan, Z.; Préhaud, C.; Cordier, F.; Simenel, C.; Delepierre, M.; Buc, H.; Lafon, M., et al. Interference with the PTEN-MAST2 Interaction by a Viral Protein Leads to Cellular Relocalization of PTEN. *Science Signaling* **2012**, *5*, ra58-ra58, doi:10.1126/scisignal.2002941.
8. Sharma, S.C.; Rupasinghe, C.N.; Parisien, R.B.; Spaller, M.R. Design, Synthesis, and Evaluation of Linear and Cyclic Peptide Ligands for PDZ10 of the Multi-PDZ Domain Protein MUPP1. *Biochemistry* **2007**, *46*, 12709-12720, doi:10.1021/bi7008135.
9. Lo Cascio, E.; Toto, A.; Babini, G.; De Maio, F.; Sanguinetti, M.; Mordente, A.; Della Longa, S.; Arcovito, A. Structural determinants driving the binding process between PDZ domain of wild type human PALS1 protein and SLiM sequences of SARS-CoV E proteins. *Comput Struct Biotechnol J* **2021**, *19*, 1838-1847, doi:10.1016/j.csbj.2021.03.014.
10. Fournane, S.; Charbonnier, S.; Chapelle, A.; Kieffer, B.; Orfanoudakis, G.; Travé, G.; Masson, M.; Nominé, Y. Surface plasmon resonance analysis of the binding of high-risk mucosal HPV E6 oncoproteins to the PDZ1 domain of the tight junction protein MAGI-1. *Journal of Molecular Recognition* **2010**, *24*, 511-523, doi:10.1002/jmr.1056.
